# Functionalization of Graphene Oxide and its Composite with Poly(3,4-ethylenedioxythiophene) as Electrode Material for Supercapacitors

**DOI:** 10.1186/s11671-015-1078-x

**Published:** 2015-09-22

**Authors:** Minchao Wang, Ruxangul Jamal, Yujie Wang, Lei Yang, Fangfang Liu, Tursun Abdiryim

**Affiliations:** Key Laboratory of Oil and Gas Fine Chemicals, Educational Ministry of China, College of Chemistry and Chemical Engineering, Xinjiang University, Urumqi, 830046 People’s Republic of China; Key Laboratory of Functional Polymers, Xinjiang University, Urumqi, 830046 People’s Republic of China; Key Laboratory of Petroleum and Gas Fine Chemicals, Educational Ministry of China, School of Chemistry and Chemical Engineering, Xinjiang University, Urumqi, 830046 People’s Republic of China

**Keywords:** PEDOT, Thiophene-grafted graphene oxide, Composite, Electrochemical performance

## Abstract

In this study, poly(3,4-ethylenedioxythiophene)/thiophene-grafted graphene oxide (PEDOT/Th-GO) composites from covalently linking of Th-GO with PEDOT chains were prepared via in situ chemical polymerization with different weight percentage of Th-GO ranging between 40 and 70 % in reaction medium. The resulting composite materials were characterized using a various analytical techniques. The structural analysis showed that the composites displayed a higher degree of conjugation and thermal stability than pure PEDOT, and the weight percentage of Th-GO could affect the doping level, amount of undesired conjugated segments, and porous structure of composites. Electrochemical analysis suggested that the highest specific capacitance of 320 F g^−1^ at a current density of 1 A g^−1^ with good cycling stability (capacitance retention of 80 % at 1 A g^−1^ after 1000 cycles) was achieved for the composite prepared from 50 wt% Th-GO content in reaction medium.

## Background

Supercapacitors, aimed at narrowing the gap between batteries and capacitors to form fast charging energy storage devices of intermediate specific energy, have attracted growing attention because of its high power density, fast charging-discharging processes and long cycle life [1, 2]. The main materials used for supercapacitor electrode preparation include carbon materials, transition metal oxides, and conducting polymer [3]. Among carbon materials, graphene, a two-dimensional carbon material, has been widely applied in supercapacitors due to its very large surface area, excellent electrical conductivity, and strong mechanical strength [4, 5]. Graphene oxide (GO), the oxidation form of graphene [6], contains oxygen functional groups such as epoxide and hydroxyl on the basal planes and carboxylic acid and other carbonyl group on the edges of the graphene nanosheets [7]. And the carbon atoms located on the edges of the GO nanosheets are other active sites, which can be used for chemical functionalization [8], and GO exhibits super ionic conductivity and high proton conductivity [9]. Furthermore, various substances have been incorporated with GO layers, including carbon nanotube, conducting polymers, and metal oxides to get synergistic contribution from hybridization to fabricate supercapacitor [10]. Poly(3,4-ethylenedioxythiophene) (PEDOT), as a most promising conducting polymer, is widely used as electrode materials for supercapacitor due to its good electrical conductivity, large pseudo capacitance, and environmental stability [11, 12]. For instance, Chen et al. reported the PEDOT/Graphene composite having specific capacitance of 257 F g^−1^ prepared by vapor-phase polymerization method [13], and Lee et al. reported PEDOT/Graphene composite prepared by electrochemical deposition method having specific capacitance of 154 F g^−1^ [14]. However, graphene tends to agglomerate irreversibly and is difficult to well re-dispersed in organic solvents and polymer matrix. For GO, although it can be readily disperses in water, the organic solvents are hard to penetrate into the interlayer spaces of GO [15]. Thus, conducting polymer would suffer from the weak adhesion and poor coverage when coated on the GO substrates. Therefore, the uniform combination of GO and conducting polymer is the current interest. It was found that covalent functionalization of GO is a promising method for grafting polymer chain onto GO as well as improving the dispersibility and interfacial interaction between polymer matrix and GO [16].

In this work, the functionalized GO (thiophene-grafted GO) was synthesized by Suzuki coupling reaction of 4-iodophenyl functionalized GO with 3-thienylboronic acid. Subsequently, the thiophene-grafted GO (Th-GO) was covalently linked with PEDOT chains via in situ polymerization of EDOT (3,4-ethylenedioxythiophene) monomer to yield PEDOT/Th-GO. Since the covalently surface functionalization (or grafting) of GO with small molecules can reduce the GO to a certain extent, the compatibility of GO with PEDOT matrix then can be improved. Furthermore, the covalently linking of Th-GO with PEDOT chains can be realized by subsequent in situ polymerization of Th-GO in the presence of EDOT monomer. Especially, 3-position of the thiophene unit is linked with GO in Th-GO, and it can promote the coupling of EDOT monomer in the 2,5-position of thiophene unit with the reducing of the undesirable coupling. The polymer growth on the surface of the Th-GO interferes with the strong π–π stacking, which can reduce Th-GO agglomeration. Based on these considerations, we took systematic studies on the structure and electrochemical properties of PEDOT/Th-GO composite with the loading of different weight percentage of thiophene-grafted GO ranging from 40 to 70 % in reaction medium. The potential application of these composite materials in high-performance supercapacitors was evaluated.

## Methods

### Materials

Graphene oxide was obtained from Nanjing XFNano Material Tech Co., Ltd (Nanjing, China). 3,4-ethylenedioxythiophene (EDOT), 3-thienylboronic, 4-iodoaniline, and cetyltrimethylammonium bromide (CTAB) were obtained from Shanghai Aladdin Reagent Company (Shanghai, China) and stored in a refrigerator prior to use. Tetrahydrofuran (THF) was purified by common methods and used immediately after purification. All other reagents were used as received without further purification.

### Synthesis of 4-Iodophenyl Functionalized GO

4-Iodophenyl functionalized GO (I-GO) was prepared as previously reported [17]. Dispersed in 200 ml concentrated sulfuric acid at room temperature and sonicated for 30 min were 100 mg of graphene oxide. Then, 4-iodoaniline (3.6 g, 16 mmol) and sodium nitrite (1.39 g, 20 mmol) were added to the reaction medium which was left under nitrogen and vigorously magnetically stirred at 60 °C for 1 h. After cooling to room temperature, the resulting liquid was poured into DMF solution and washed with DMF until the filtration was colorless. The product was finally washed with ethanol and dried in a vacuum oven (60 °C) for 24 h.

### Synthesis of 4-Thienylphenyl Functionalized GO (Th-GO, Thiophene-Grafted Graphene Oxide)

4-Thienylphenyl functionalized GO (Th-GO, thiophene-grafted graphene oxide) was synthesized via Suzuki coupling reaction [18, 19]. I-GO (50 mg) was dissolved in 13 ml THF, followed by addition of 3-thienylboronic (28 mg, 0.21 mmol), and Na_2_CO_3_ (45 mg, 0.42 mmol) in distill water (370 mg) at room temperature. This reaction mixture was then degassed by three freeze-pump-thaw cycles and refilled with nitrogen. Pd(PPh_3_)_4_ (12 mg, 0.01 mmol) was quickly added to the reaction mixture and stirred at 70 °C for 72 h. After cooling to temperature, the mixture was filtered, and the product was washed thoroughly with THF, methanol, water, methanol, and THF. Finally the product Th-GO was dried under vacuum (60 °C) for 24 h.

### Preparation of the Composites and PEDOT

To a 100-ml flask charged with Th-GO (0.1 g), cetyltrimethylammonium bromide (CTAB, 0.16 g, 0.01 M of final concentration), and CHCl_3_ (40 ml), the reaction took place for 30 min under ultrasonication (40 kHz), then followed by the addition of the oxidant FeCl_3_ (0.57 g, 3.5 mmol) followed by an additional 30 min sonication. EDOT (0.1 g, 0.7 mmol) was previously dissolved in 5 ml CHCl_3_ and added dropwise to the reaction mixture. The polymerization took place for 1 h. Finally, the product was washed repeatedly with water and methanol until the filtrate was colorless, and then the power dried under vacuum at 60 °C for 48 h. The composite was denoted as PEDOT/Th-GO, which was prepared from 50 wt% Th-GO. The other PEDOT/Th-GO from different weight percentage of Th-GO were prepared in similar manner by adjusting the weight percentage of Th-GO as 40, 60, and 70 wt%.

For comparison, the pure PEDOT was also synthesized in a similar manner without Th-GO. The content of Th-GO and EDOT in reaction medium is listed in Table 1.

**Table 1:** The content of Th-GO and EDOT in reaction medium

Sample	Monomer (g)	CTAB (g)	FeCl_3_ (g)	Th-GO (g)
PEDOT	0.1	0.16	0.57	0
40 wt% Th-GO	0.1	0.16	0.57	0.067
50 wt% Th-GO	0.1	0.16	0.57	0.1
60 wt% Th-GO	0.1	0.16	0.57	0.15
70 wt% Th-GO	0.1	0.16	0.57	0.23

## Characterization Techniques

The structural and morphological characterization of PEDOT/Th-GO composites was conducted by FT-IR, UV-vis, Raman, XRD, SEM, EDS, and TGA. The Fourier transform infrared (FT-IR) spectra of the samples were obtained using a BRUKER EQUINOX-55 Fourier transform infrared spectrophotometer (Bruker, Billerica, MA, USA) (frequency range 4000 to 500 cm^−1^). UV-vis absorption spectra of the samples were recorded on a UV-vis spectrophotometer (UV4802, Unico, Dayton, NJ, USA). The Raman shift spectra were carried out in a backscattering geometry with the 1064 nm excitation wavelength using a Bruker Vertex 70 FT Infrared Spectrometer (equipped with RamIIFT Raman Module). XRD analysis was conducted using a Bruker AXS D8 diffractometer, and the scan range (2*θ*) was 10° to 80°, with monochromatic CuKα radiation source (*λ* = 0.15418 nm). SEM was investigated by using Hitachi S-4800 field emission scanning electron microscope. The elemental contents of samples were characterized by energy dispersive spectroscopy (EDS), which was taken on a Leo1430VP microscope with operating voltage 5 kV. Thermogravimetric analysis (TGA) was carried out using a NETZSCH Simultaneous Thermal Analyzer STA 409 PC Luxx under a nitrogen atmosphere with a heating rate of 10 °C min^−1^.

## Electrochemical Test

All electrochemical experiments were carried out using a three-electrode system by using CHI 660C electrochemical workstation (CH Instruments Inc., Shanghai, China). The sample was used as the working electrodes, platinum as the counter electrode, saturated calomel electrode (SCE) as reference electrode, and 1 M H_2_SO_4_ was used as electrolyte. The cyclic voltammetry (CV), galvanostatic charge-discharge (GCD) tests, and cycle stability were done in the potential window ranging from −0.2 V to 0.8 V. And the GCD and cycle stability were tested at current densities of 1 A g^−1^, and the electrochemical impedance spectroscopy (EIS) tests were carried out in the frequency range from 0.01 Hz to 100 KHz at open-circuit potential with an ac perturbation of 5 mV. The working electrodes of electrochemical capacitors were formed by mixing 85 wt% active materials (3 mg), 10 wt% carbon black, and 5 wt% polytetrafluoroethylene (PTFE) to form slurry. The slurry was pressed on graphite current collectors with an area of 1 × 1 cm^2^ to form electrodes then dried at 60 °C for 24 h.

## Results and Discussion

**Fig. 1:**
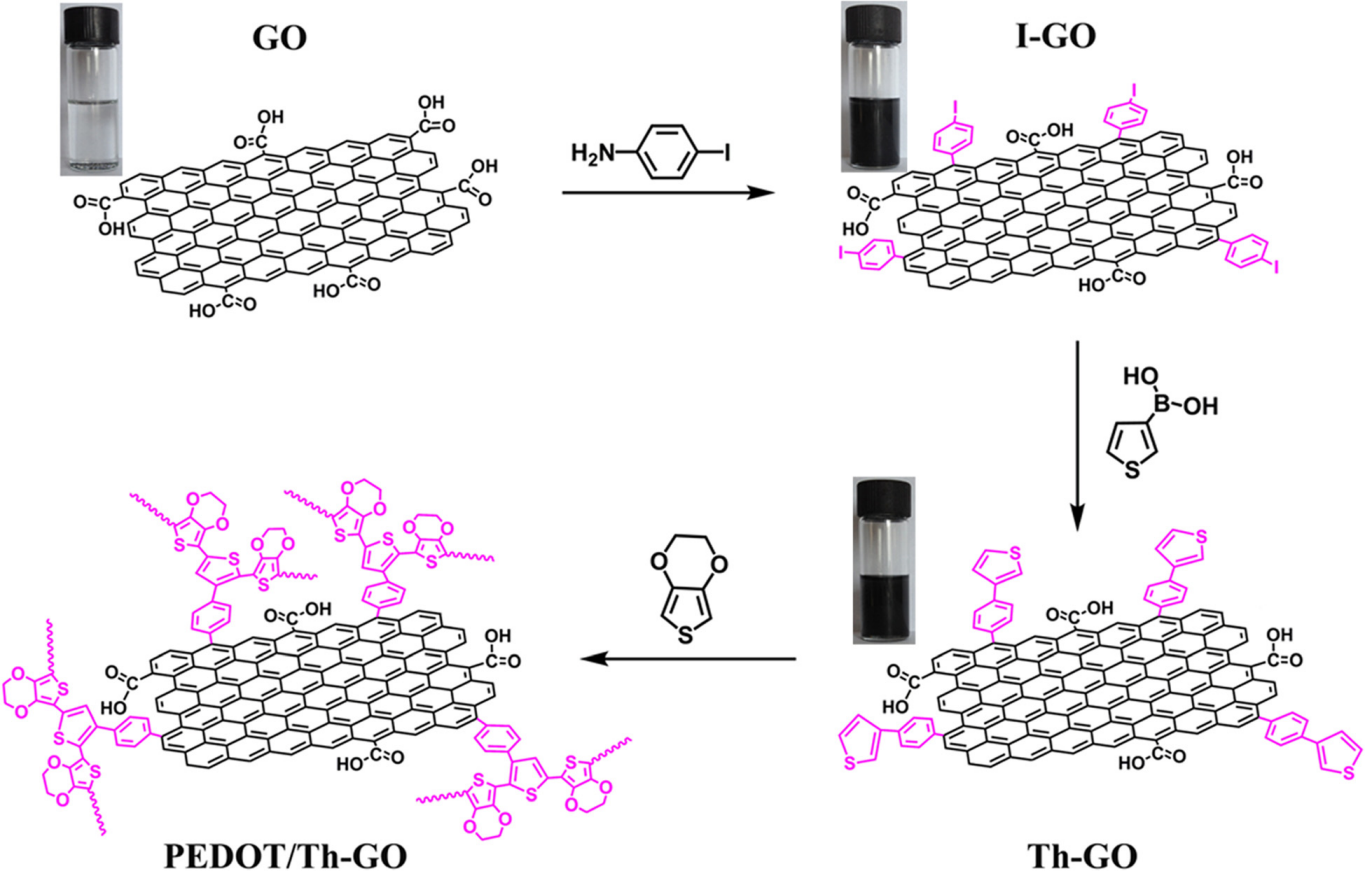
The schematic for the fabrication of PEDOT/Th-GO composites

The synthesis route of PEDOT/Th-GO composite is schematically shown in Fig. 1. As depicted in Fig. 1, the functionalization of GO has two steps: due to many defects at the edges of GO which can provide active sites for chemical reactions, the diazonium radicals can easily graft to the active sites on the GO surface by covalent linkages [8]. The first step is functionalization of GO through the reaction between GO and 4-iodoaniline to form 4-iodophenyl functionalized GO [20]. The second step is grafting of thiophene groups to GO, which can be realized by Suzuki coupling reaction between 4-iodophenyl functionalized GO (I-GO) and thiophene. As shown in inset of Fig. 1, the GO cannot be dispersed in CHCl_3_ to form a stable colloidal suspension, while a stable colloidal suspension of I-GO and Th-GO in CHCl_3_ obtained in the case of grafted GO. This phenomenon further proves that the covalently surface functionalization (or grafting) of GO with small molecules (4-iodophenyl or 4-thienylphenyl) can reduce the GO to a certain extent, and this can enhance the dispersion of functionalized GO in organic solvent, which can avoid an inevitable aggregation or the restacking of graphene sheets in the polymer/graphene composites [21].

**Fig. 2:**
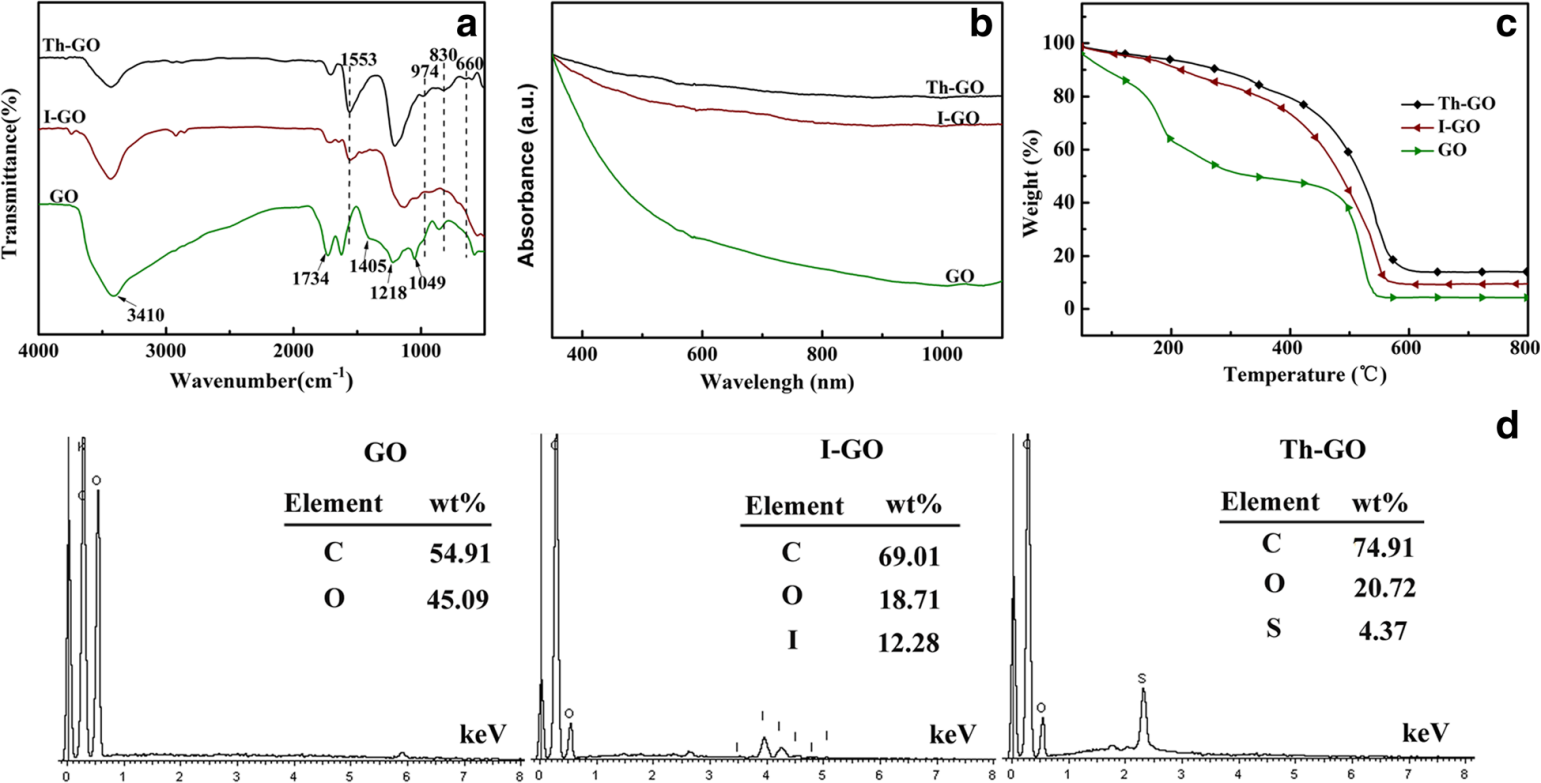
**a** FT-IR spectra, **b** UV-vis spectra, **c** TGA thermograms, and **d** EDS of GO, I-GO and Th-GO

The structure characterization of synthesized I-GO and Th-GO is depicted in Fig. 2. Figure 2a represents the FT-IR spectra of GO, I-GO, and Th-GO. The characteristic vibration bands of GO are observed at 3410 (O − H), 1734 (C = O), 1405 (O − H), 1218 (C–O), and 1049 cm^−1^ (C–O), which are originated from hydroxy, carboxy, epoxy, and alkoxy groups of GO, respectively [22]. Comparing with that of GO, the decrease in relative intensity of vibration bands from O–H (at 3410 cm^−1^) and C = O (at 1734 cm^−1^) in the case of I-GO implies that the GO is reduced by 4-iodoaniline. The new peak at 1553 cm^−1^ is attributed to the skeletal vibration of the benzene ring [23]. These results clearly indicate that the p-iodophenyl groups are successfully grafted into the GO surface. For Th-GO, the additional vibration bands at 974, 830, 660 cm^−1^ are originated from the C–S–C bond in the thiophene ring [24], indicating the thiophene groups are successfully grafted into the GO. Figure 2b shows the UV-vis absorption spectra of GO, I-GO, and Th-GO. As shown in Fig. 2b, the UV-vis absorbance of I-GO and Th-GO increases significantly when compared to GO, which indicates the efficient reduction of GO and restores π–π conjugations [5]. It further confirms that the p-iodophenyl and thiophene groups are well bound onto GO surface. Figure 2c shows the TGA of GO, I-GO, and Th-GO. The GO reveals two evident mass losses, which are resulted from absorbed water and pyrolysis of oxygen-containing groups [25, 26]. Furthermore, to compare with GO, the I-GO and Th-GO displayed better thermal stability, suggesting that the benzene or thiophene groups in I-GO and Th-GO can promote the formation of heat-stable structure [20]. GO, I-GO, and Th-GO were also analyzed by EDS (Fig. 2d). The percentage contents of the major elements, C and O, were 54.91 and 45.09 and 69.01 and 18.71 for GO and I-GO, respectively. The ratios of percentage contents of C and O elements for GO and I-GO are 1.21 and 3.69, respectively, which indicates that the deoxygenation of GO takes place during the functionalization process. And the presence of I element in I-GO indicates that the p-iodophenyl is successfully bound to GO. Comparing with that of I-GO, the presence of S element in Th-GO indicates the incorporation of thiophene unit in the GO structure. Based on these analyses from FT-IR spectra, UV-vis spectra, TGA, and EDS, it can be concluded that the p-iodophenyl and thiophene are successfully grafted to GO.

**Fig. 3:**
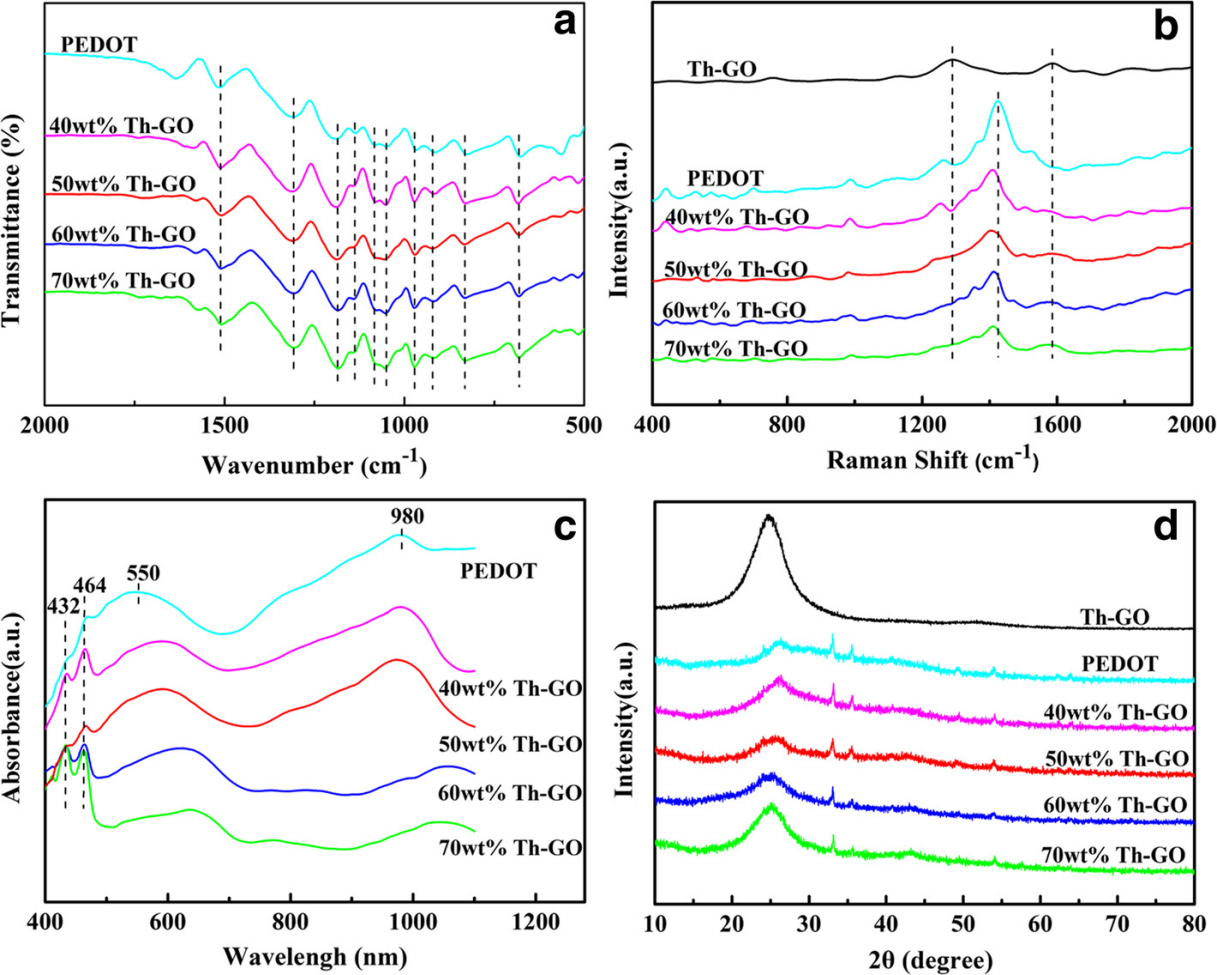
**a** FT-IR spectra, **b** Raman spectra, **c** UV-vis spectra, and **d** XRD patterns of Th-GO, PEDOT, and PEDOT/Th-GO composites

Figure 3a shows the FT-IR spectra of PEDOT and composites. For PEDOT, the bands at 1515 and 1309 cm^−1^ are assigned for the vibration of C = C and C–C bonds, respectively, while the vibration bands at 1189, 1137, 1088, and 1048 cm^−1^ originate from the vibration of C–O–C bond, and the vibration bands at 974, 920, 830, and 660 cm^−1^ are assigned to the vibration of C–S–C bond [24, 27]. It is obvious that all the spectra of composites are identical to each other for the loading of different weight percentage of Th-GO ranging from 40 to 70 % in reaction medium, and the characteristic bands of Th-GO and PEDOT are also found in the spectra of the composites.

The Raman spectra of PEDOT and composites are presented in Fig. 3b. For Th-GO, two typical bands for D- and G-band are observed at 1303 and 1595 cm^−1^, respectively, which are the intrinsic features of GO [28]. For PEDOT, the characteristic bands at 1519 and 1424 cm^−1^ are attributed to C = C stretching, while C–C inter-ring and C–O stretching bands are observed at 1256 and 1111 cm^−1^, respectively, and the oxyethylene ring and C–S–C deformation bands are observed at 989 and 707 cm^−1^ [29, 30]. Comparing with PEDOT, although the D-band from Th-GO almost overlapped by the band of PEDOT in composites, the relative intensity of G-band (1595 cm^−1^) increases with increase of weight percentage of Th-GO, and the bands of PEDOT in composite shift to low frequency, indicating a strong interaction between PEDOT and Th-GO composites resulted from the π–π stacking interactions between PEDOT and GO [2]. Based on the results from FT-IR and Raman spectra, it can be deduced that the Th-GO has been successfully incorporated with the PEDOT.

Figure 3c shows the UV-vis absorption spectra of PEDOT and composites in 1-methyl-2-pyrrolidinone(NMP). As shown in Fig. 3c, the composites and the pure PEDOT display two main broad absorption peaks approximately at 500 to 700 nm and at 950 to 1050 nm with a free tail extending into the near-infrared region. In addition, both PEDOT and composites have two minor absorption peaks at 432 and 464 nm. The peaks approximately at 500 to 700 nm can be ascribed to the π–π* transition [31], and the peaks approximately at 950 to 1050 nm can be associated to polaron and/or bipolaron bands originating from high conjugation length [32]. The peaks at 432 and 464 nm for both of PEDOT and composites are assigned to the conjugated segments having different conjugation lengths [33], and it is generally believed that these conjugated segments play negative effect on the improving of electrochemical activity of polymer matrix. To compare with that of pure PEDOT, the redshift in π–π* transition peaks of composites from 550 to 600 and 627 nm indicates that the composites have higher degree of conjugation than PEDOT. Moreover, it is clear from Fig. 3c that the degree of conjugation of the composites increases with an increase in Th-GO content. On the contrary, the intensity ratio of the polaron and/or bipolaron peak to the π–π* transition peak (*I*_950–1050_/*I*_500–700_) of the composites decreases with an increase in Th-GO content, suggesting that the doping level of composites decreases with an increase in Th-GO content [34]. Besides, the relative intensity ratio of the peaks from conjugated segments (at 432 and 464 nm) to the π–π* transition peak (approximately at 500 to 700 nm) is the lowest in composite from 50 wt%, which will be helpful in enhancing the electrochemical activity of polymer matrix.

Figure 3d presents the XRD patterns of Th-GO, PEDOT, and composites. The Th-GO shows a strong diffraction peak at 2*θ* = 24.8°, while the pure PEDOT has a broad diffraction peak at 2*θ* = 26.3°, and it is associated to the intermolecular π–π stacking [12]. In addition, the sharp diffraction peaks with low intensity at 2*θ* = 33°, 35°, 49°, and 54° in PEDOT and composites correspond to the doping agent of FeCl_4_^−^ [35]. To compare with that of PEDOT, the main diffraction peaks (π–π stacking) of composites shift from 2*θ* = ~26° to 2*θ* = ~25° with the increase of weight percentage of Th-GO in composite, and the gradual increase in relative intensity of diffraction peaks between 2*θ* = 25.5° and 2*θ* = 26.3° is also observed with the increase of weight percentage of Th-GO in composite. All these observations indicate that the composites are not the simple physical mixture of Th-GO and PEDOT, and the interaction between Th-GO and PEDOT increases with weight percentage of Th-GO [35, 36].

**Fig. 4:**
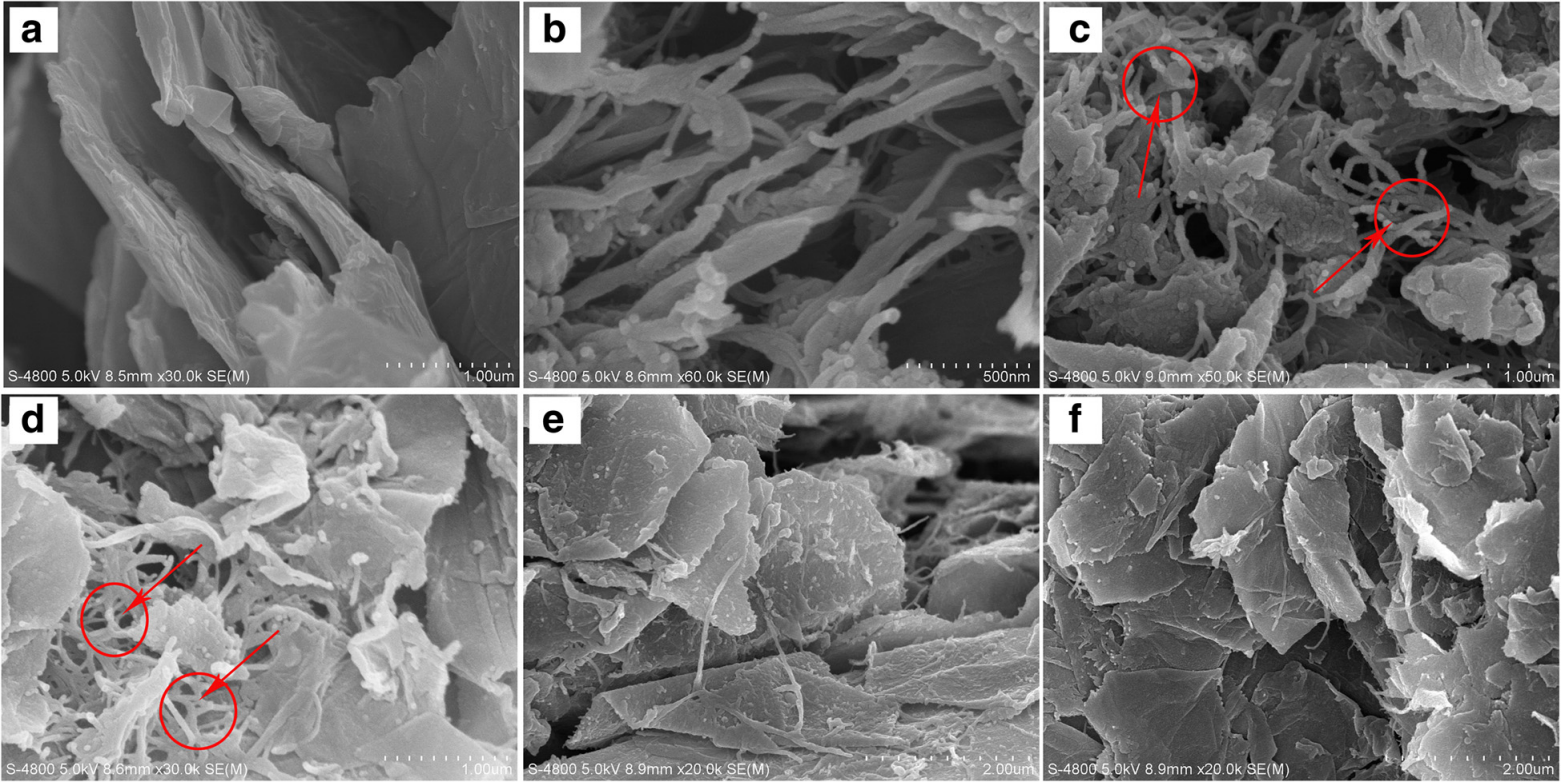
SEM images of **a** Th-GO, **b** PEDOT, **c** 40 wt% Th-GO, **d** 50 wt% Th-GO, **e** 60 wt% Th-GO, **f** 70 wt% Th-GO

Figure 4 shows the SEM images of Th-GO, PEDOT, and composites. The SEM image in Fig. 4a displays a rather regular and smooth flake structure of Th-GO. While PEDOT shows the 50–100 nm thick nanofibers (Fig. 4b). This is because the PEDOT chain spreads very fast in a limited time and transforms into many initial nanofibrous oligomers at the beginning stage [37]. As the polymerization time grows, EDOT species and the newly formed nanofibrous oligomers can be used as a soft template, which will form the nanofibrous structures [37]. And after incorporation of Th-GO, the composites show that numerous interconnected PEDOT nanofibers grow on the Th-GO surface. Comparing with Th-GO, the composites show a considerably rough and irregular surface, which further indicates that Th-GO is wrapped by PEDOT. And more porous structure (indicated by arrows in Fig. 4c, d) occurs in composites prepared from 40 and 50 wt% Th-GO in reaction medium than other composites, and this porous structure will provide broader space and shorter diffusion distance for ion transport between the electrolyte and composite. However, the lower Th-GO content can cause PEDOT nanofibers adjoin or grow together to form agglomerate, and this will lead to decrease in uniform covering of Th-GO, consequently reducing the π–π interaction of Th-GO and PEDOT.

**Fig. 5:**
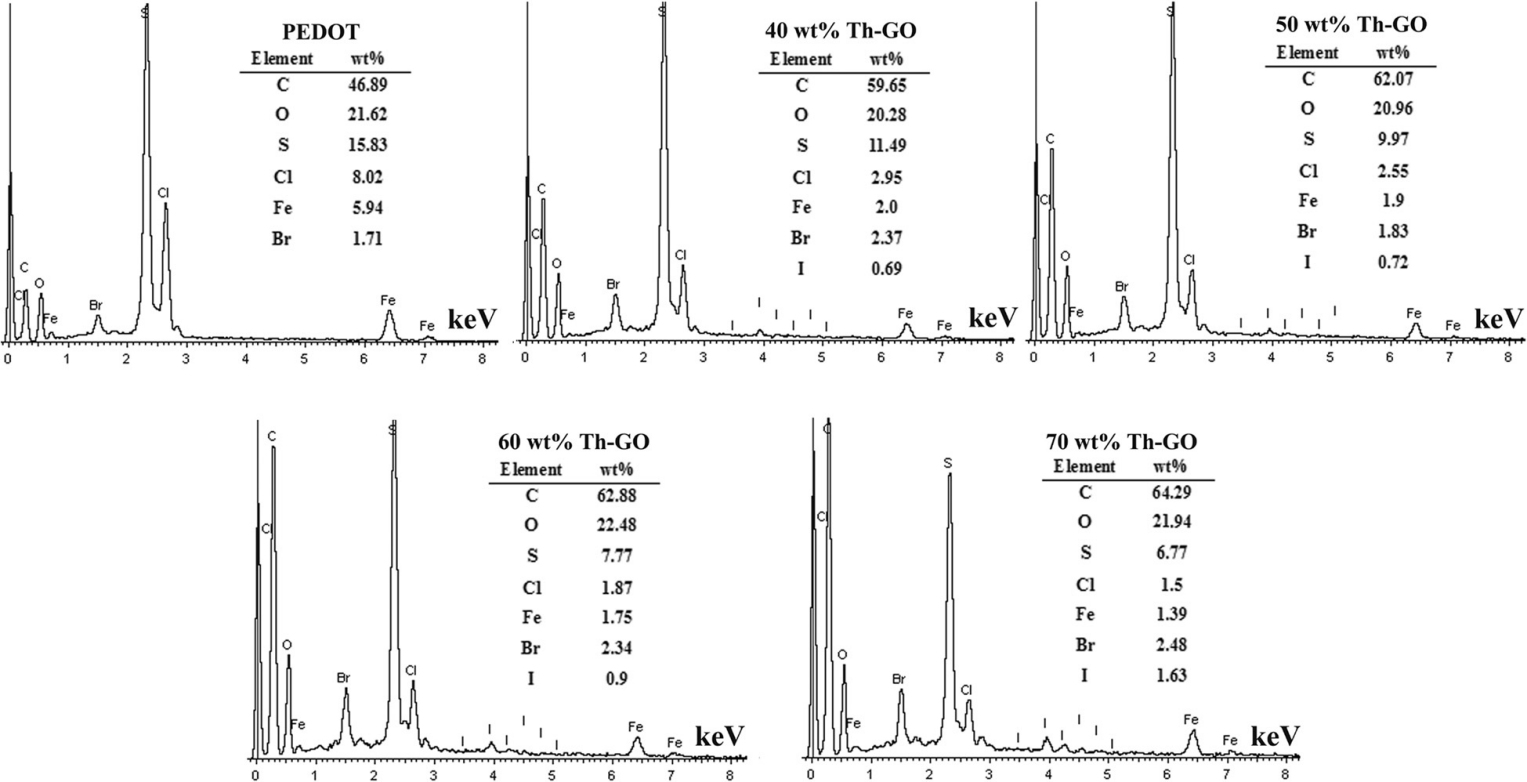
EDS of PEDOT and PEDOT/Th-GO composites

Figure 5 displays the EDS of PEDOT and composites. As seen in Fig. 5, the Cl, Fe, and Br elements of PEDOT originated from the doping agent of FeCl_4_^−^ and CTAB. The decrease in weight percentage of S element is observed by increasing the weight percentage of Th-GO, indicating a decrease in polymer weight in composites.

**Fig. 6:**
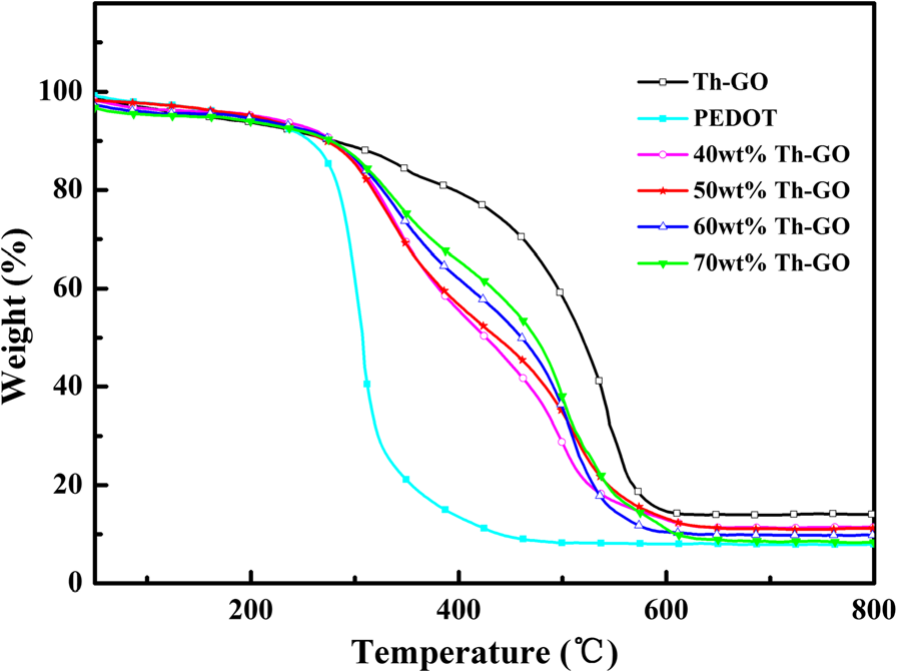
TGA thermograms of Th-GO, PEDOT, and PEDOT/Th-GO composites

Figure 6 shows the TGA analysis for Th-GO, PEDOT, and composites. Figure 6 reveals that Th-GO, PEDOT, and composites undergo a 5 % weight loss at above 150 °C (Th-GO), 189 °C (PEDOT), 202 °C (40 wt% Th-GO), 203 °C (50 wt% Th-GO), 185 °C (60 wt% Th-GO), and 170 °C (70 wt% Th-GO). As shown in Fig. 6, the composites display three major weight-loss steps. The first weight-loss step (less than 5 %) corresponds to the volatilization of solvents and/or absorbed moisture and oligomers of composites [35]. The second weight-loss step (170–203 to 436–462 °C) assigned partly to the weight loss of ethylenedioxyl in PEDOT, the oxygen-containing groups of Th-GO, and partly to thermal decomposition of composites matrix [38]. The third weight-loss step (436–462 to 800 °C) is ascribed to the weight loss of thermal decomposition of composites matrix [39]. The Th-GO, PEDOT, and composites (from 40 to 70 wt% Th-GO) final residue at 800 °C are 14.1, 8.1, 11.2, 11.1, 9.8, and 8.6 wt%. Comparing with PEDOT, the composites exhibit better thermal stability, which is the result of strong interaction between Th-GO and PEDOT. While the composites prepared from 60 and 70 wt% Th-GO show lower residue which is due to their higher amounts of undesired conjugated segments (at 432 and 464 nm), thermal decomposed easily in high temperature. The excellent thermally stable composite is obtained in the case of composite prepared from 50 wt% Th-GO, which undergoes a 5 % weight loss at above 203 °C with the final residue of 11.1 wt%.

**Fig. 7:**
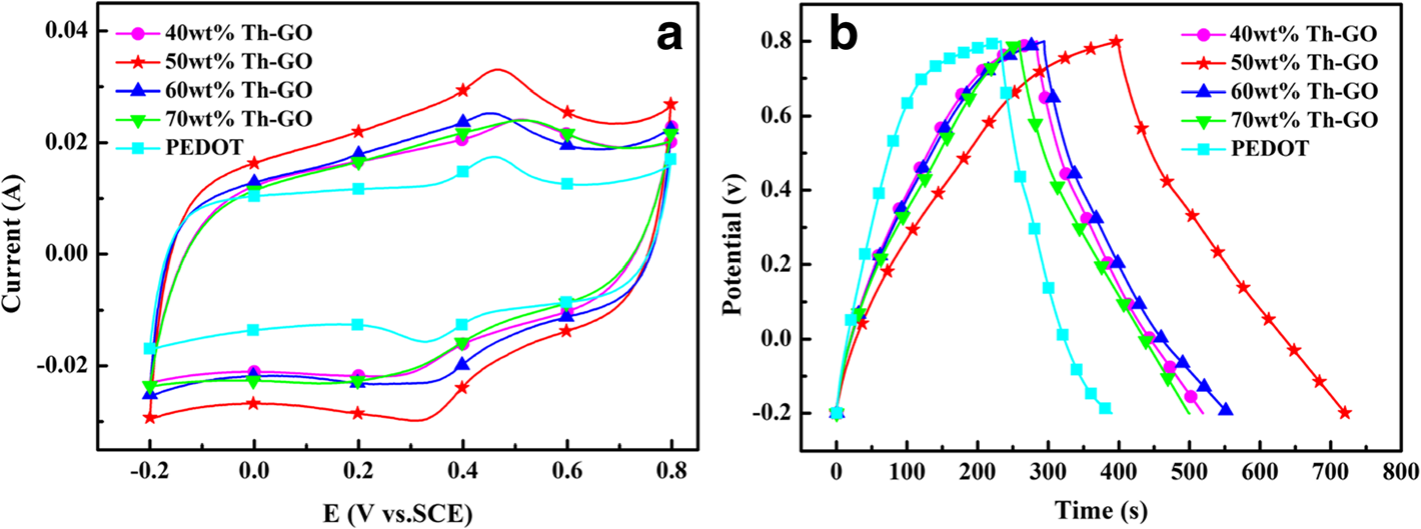
Electrochemical performances of PEDOT and composites in 1 M H_2_SO_4_. Mass of the active material, 3 mg; **a** CV curves at scan rate of 50 mV s^−1^; **b** Galvanostatic charge-discharge curves at current density of 1 A g^−1^

Figure 7a represents the CV curves of the PEDOT and composites at a scan rate of 50 mV s^−1^. Furthermore, it is observed that at the same scan rate, the 50 wt% Th-GO composite has the biggest current response than others, indicating that the composite prepared from 50 wt% Th-GO has the excellent capacitance behavior. Figure 7b represents the galvanostatic charge-discharge (GCD) curves of the PEDOT and composites at current densities of 1 A g^−1^. The specific capacitance (SC) of the electrode material can be calculated from the GCD curves according to the following equation: SC = (*I* × Δ*t*)/(Δ*V* × *m*) [40], where SC is the specific capacitance (F g^−1^), Δ*V* represents the sweep potential range (1 V), and *m* is the mass loading of the active material within the electrode (3 mg). *I* denotes the response current density, and Δ*t* is the discharge time. On the basis of the GCD curves, the specific capacitances of PEDOT and composites are 155 F g^−1^ (PEDOT), 236 F g^−1^ (40 wt% Th-GO), 320 F g^−1^ (50 wt% Th-GO), 260 F g^−1^ (60 wt% Th-GO), and 240 F g^−1^ (70 wt% Th-GO). It is clear that the composites have higher SC than PEDOT, and the composite prepared from 50 wt% Th-GO shows the highest SC of 320 F g^−1^, which is higher than that of previously reported PEDOT/graphene composite [13].

As concluded from FT-IR spectra of I-GO, the deoxygenation of GO takes place during the functionalization process, which can facilitate the electron transfer and bring a synergistic effect by an increase of the π–π stacking interaction between GO and PEDOT [41]. Furthermore, the covalently linking of Th-GO and PEDOT also enhances the π–π interaction between Th-GO and PEDOT chains, improving the capacitance of composite. It should be noted that the covalently surface functionalization (or grafting) of GO with small molecules can avoid an inevitable aggregation or the restacking of graphene sheets in the polymer/graphene composites [21], which is also a benefit for the π–π interaction to improve electrochemical properties composite. As discussed above, the composites prepared from 40 wt% Th-GO have porous structure, which can enhance the capacitive behavior. However, the highly agglomeration of PEDOT nanofibers also occurs in the composite prepared from 40 wt% Th-GO, which plays negative effect on the capacitive behavior composite. It is clear from UV-vis analysis that the PEDOT in composites prepared from 60 and 70 wt% Th-GO has high conjugation lengths with low doping level. However, the high amount of undesired conjugated segments (at 432 and 464 nm) in composites prepared from 60 to 70 wt% Th-GO play negative effect on the electrochemical activity of composites. Therefore, the composites prepared from 40 and 70 wt% Th-GO display lower SC than the other composites, while the composite prepared from 50 wt% Th-GO with the porous structure, high doping level and low amount of undesired conjugated segments can display the highest SC among composites.

**Fig. 8:**
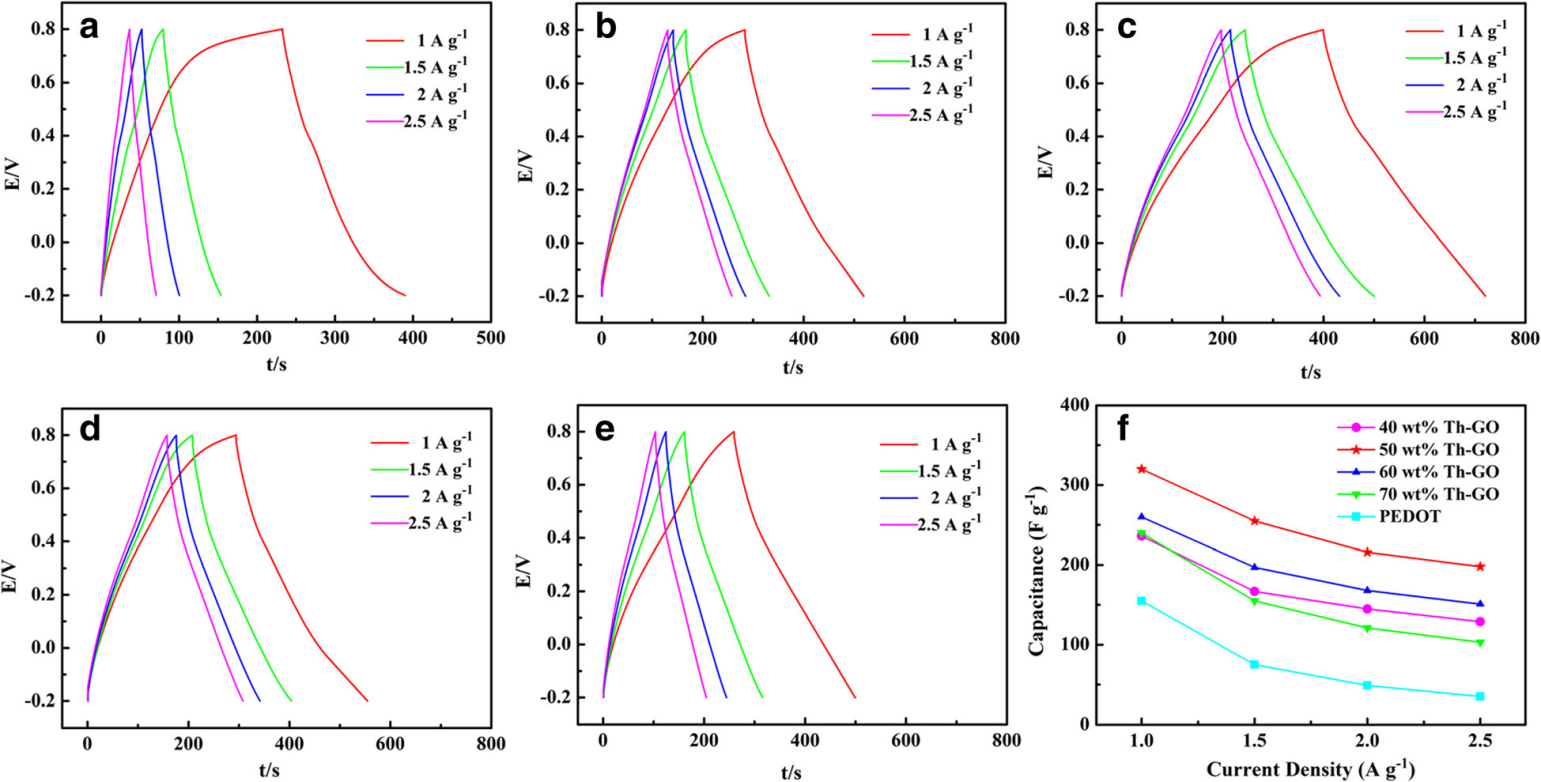
Charge-discharge curves of PEDOT and composites at the current density of 1, 1.5, 2, and 2.5 A g^−1^ in 1 M H_2_SO_4_. Mass of the active material, 3 mg. **a** PEDOT, **b** 40 wt% Th-GO, **c** 50 wt% Th-GO, **d** 60 wt% Th-GO, **e** 70 wt%, **f** plots of corresponding specific capacitance as a function of current density

Figure 8 shows the GCD curves of PEDOT and composites at the current density range from 1–2.5 A g^−1^. It can be seen that all composite electrodes exhibit an almost equilateral triangle shape and the potential time relationships are all approximately linear, indicating a good reversibility during the charge-discharge processes and exhibiting excellent capacitive behavior. Obviously, with the current density increasing, all the electrode materials’ specific capacitance decreased. Furthermore, the composites show a higher specific capacitance than PEDOT, and the 50 wt% Th-GO composite achieve the highest specific capacitance of 320, 255, 216, and 198 F g^−1^ at current densities of 1, 1.5, 2, and 2.5 A g^−1^. The result indicates that 50 wt% Th-GO composite has a good specific capacitance even at a high current density.

**Fig. 9:**
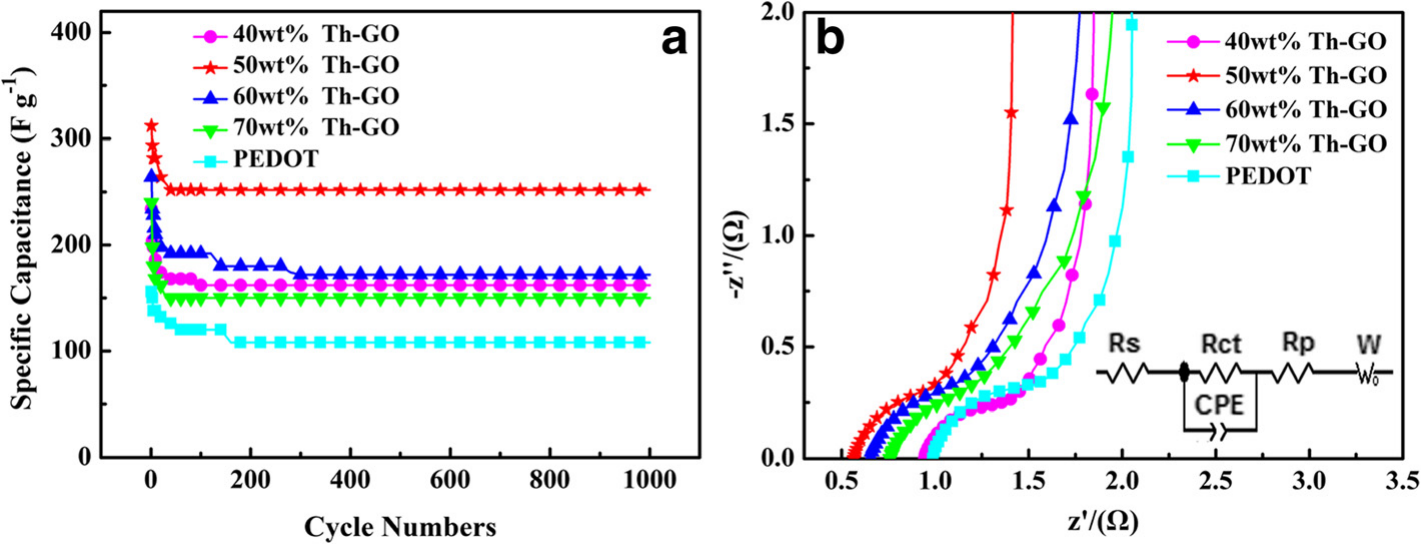
**a** Specific capacitances as a function of cycle number measured at 1 A g^−1^; **b** EIS curves at 5 mV over the frequency range of 0.01–100 KHz

Figure 9a shows the SC values of PEDOT and composites as functions of the number of consecutive charge-discharge cycles. As shown in Fig. 9a, a steady state is observed in PEDOT after 160 cycles, and the composites display steady state after 90 cycles (40 wt% Th-GO), 40 cycles (50 wt% Th-GO), 280 cycles (60 wt% Th-GO), and 30 cycles (70 wt% Th-GO). Moreover, the capacitance retention of PEDOT and composites are 69 % (PEDOT), 68 % (40 wt% Th-GO), 80 % (50 wt% Th-GO), 66 % (60 wt% Th-GO), and 63 % (70 wt% Th-GO). Thus, it can be concluded that the composites can easily reach to steady state except for the composite prepared from 60 wt% Th-GO. It should be noted that the strong mechanical strength of GO should reduce the volumetric change of composite during the charge-discharge cycles, which will be beneficial for the improvement of the cycle stability and capacity retention of composite. However, the capacity retention of composites during the charge-discharge cycles from the initial state to steady state is lower than that of PEDOT, except the composite prepared from 50 wt% Th-GO. These variations in cycling stability can be assigned to the structural differences of composites as mentioned above. The excellent cycling stability with the highest capacity retention occurs in the case of composite prepared from 50 wt% Th-GO, which is originated from the porous structure, high doping level, and low amount of undesired conjugated segments as well as the uniform combination of Th-GO and PEDOT in this composite.

Figure 9b shows the Nyquist plots of PEDOT and composites. The equivalent circuit model is shown in the inset of Fig. 9b. All Nyquist plots are semicircular over the high-frequency range and linear in the low-frequency region. And the Nyquist plots can be well-fit to an equivalent circuit. At high frequency, the intersection point on the real axis represents the internal resistance (*R*_s_), and the semicircular behavior corresponds to the parallel connection of the interfacial charge-transfer resistance (*R*_ct_) and constant phase element (CPE, double-layer capacitance), while low-frequency region is the sum of *R*_p_ (capacitor resistance) and the W (Warburg impedance). Nyquist plots show the intercepts with real axis at 0.99 Ω, 0.94 Ω, 0.56 Ω, 0.65 Ω, and 0.76 Ω, which are the *R*_s_ values for the PEDOT and composites prepared from 40, 50, 60, and 70 wt% Th-GO. The *R*_ct_ estimated from the diameter of the semicircle are 0.34 Ω, 0.27 Ω, 0.28 Ω, 0.31 Ω, and 0.31 Ω for the PEDOT and composites prepared from 40, 50, 60, and 70 wt% Th-GO. Among composites, the lowest *R*_s_ value of 0.56 Ω for composite prepared from 50 wt% Th-GO indicates that this composite has the lowest internal resistance, which can be ascribed excellent synergistic contribution from hybridization of Th-GO and PEDOT. Moreover, to compare with others, the low interfacial charge-transfer resistance of composites is 0.27 and 0.28 Ω for composite prepared from 40 and 50 wt% Th-GO, respectively, which is the result of highly porous structure of these composites. Besides its low resistance, inclined straight line of composite prepared from 50 wt% Th-GO almost parallel with imaginary axis, indicating that this composite has a better capacitive behavior among the composites.

## Conclusions

In summary, the PEDOT/Th-GO composites from covalently linking of Th-GO with PEDOT chains were prepared via in situ polymerization. The systematic structural analysis revealed that the thiophene-grafted GO (Th-GO) highly dispersible in CHCl_3_ to form stable colloidal suspension, and the thiophene unit on GO promotes the covalently linking of Th-GO with PEDOT by avoiding undesirable coupling. Furthermore, to compare with PEDOT, the degree of conjugation, thermal stability, and electrochemical activity of composites were improved by the strong π–π interaction between Th-GO and PEDOT, and the variation of electrochemical properties of composites depended on the different weight percentage of Th-GO content. However, an increase in Th-GO content played negative effect on the increasing of doping level and the formation of porous structure for composites. As a result, the composite prepared from 50 wt% with the porous structure, high doping level, and low amount of undesired conjugated segments displayed the excellent capacitive behavior among composites. And the highest SC of 320 F g^−1^ at 1 A g^−1^ with capacitance retention of 80 % after 1000 cycles was achieved for the composite prepared from 50 wt% Th-GO, which suggested that the PEDOT/Th-GO could be used as a promising material for supercapacitor applications.

## References

[1] Snook GA, Kao P, Best AS (2011). Conducting-polymer-based supercapacitor devices and electrodes. Power Sources.

[2] Zhang J, Zhao XS (2012). Conducting polymers directly coated on reduced graphene oxide sheets as high-performance supercapacitor electrodes. J. Phys. Chem. C.

[3] Fan HS, Wang H, Zhao N, Xu J, Pan F (2014). Nano-porous architecture of N-doped carbon nanorods grown on graphene to enable synergetic effects of supercapacitance. Sci. Rep..

[4] Liu Y, Wang H, Zhou J, Bian L, Zhu E, Hai J, Tang J, Tang W (2013). Graphene/polypyrrole intercalating nanocomposites as supercapacitors electrode. Electrochim. Acta.

[5] Cha I, Lee EJ, Park HS, Kim J-H, Kim YH, Song C (2014). Facile electrochemical synthesis of polydopamine-incorporated graphene oxide/PEDOT hybrid thin films for pseudocapacitive behaviors. Synth. Met..

[6] Zhang K, Duan X, Zhu X, Hu D, Xu J, Lu L, Sun H, Dong L (2014). Nanostructured graphene oxide–MWCNTs incorporated poly(3,4-ethylenedioxythiophene) with a high surface area for sensitive determination of diethylstilbestrol. Synth. Met..

[7] Compagnini G, Russo P, Tomarchio F, Puglisi O, D’Urso L, Scalese S (2012). Laser assisted green synthesis of free standing reduced graphene oxides at the water–air interface. Nanotechnology.

[8] Lu Y, Jiang Y, Wei W, Wu H, Liu M, Niu L, Chen W (2012). Novel blue light emitting graphene oxide nanosheets fabricated by surface functionalization. J. Mater. Chem..

[9] Si W, Lei W, Han Z, Zhang Y, Hao Q, Xia M (2014). Electrochemical sensing of acetaminophen based on poly(3,4-ethylenedioxythiophene)/graphene oxide composites. Sens. Actuators B.

[10] Lu L, Zhang O, Xu J, Wen Y, Duan X, Yu H, Wu L, Nie T (2013). A facile one-step redox route for the synthesis of graphene/poly (3,4-ethylenedioxythiophene) nanocomposite and their applications in biosensing. Sens. Actuators B.

[11] Li W, Chen J, Zhao J, Zhang J, Zhu J (2005). Application of ultrasonic irradiation in preparing conducting polymer as active materials for supercapacitor. Mater. Lett..

[12] Alvi F, Ram MK, Basnayaka PA, Stefanakos E, Goswami Y, Kumar A (2011). Graphene–polyethylenedioxythiophene conducting polymer nanocomposite based supercapacitor. Electrochim. Acta.

[13] Chen Y, Xu J, Mao Y, Yang Y, Yang W, Li S (2013). Electrochemical performance of graphene-polyethylenedioxythiophene nanocomposites. Mater. Sci. Eng B.

[14] Lee S, Cho MS, Lee H, Nam J-D, Lee Y (2012). A facile synthetic route for well defined multilayer films of graphene and PEDOTvia an electrochemical method. J. Mater. Chem..

[15] Yang H, Li F, Shan C, Han D, Zhang Q, Niu L, Ivaska A (2009). Covalent functionalization of chemically converted graphene sheets via silane and its reinforcement. J. Mater. Chem..

[16] Deetuam C, Samthong C, Thongyai S, Praserthdam P, Somwangthanaroj A (2014). Synthesis of well dispersed graphene in conjugated poly(3,4-ethylenedioxythiophene): polystyrene sulfonate via click chemistry. Compos. Sci. Technol..

[17] Park O-K, Hahm MG, Lee S, Joh H-I, Na S-I, Vajtai R, Lee JH, Ku B-C, Ajayan PM (2012). In situ synthesis of thermochemically reduced graphene oxide conducting nanocomposites. Nano Lett..

[18] Hu X, Zuo L, Pan H, Hao F, Pan J, Fu L, Shi M, Chen H (2012). Synthesis and photovoltaic properties of n-type conjugated polymers alternating 2,7-carbazole and arylene diimides. Sol. Energy Mater. Sol. Cells.

[19] Wang L, Qing F, Sun Y, Li X, Wang H (2013). Synthesis and photovoltaic properties of poly(5,6-bis(octyloxy)-4,7-di(thiophen-2-yl)benzo-[c][1,2,5]-thiadiazole-9,9-dioctylfluorene). J. Mater. Sci. Technol..

[20] Xu LQ, Liu YL, Neoh KG, Kang ET, Fu GD (2011). Reduction of graphene oxide by aniline with its concomitant oxidative polymerization. Macromol. Rapid Commun..

[21] Eswaraiah V, Balasubramaniam K, Ramaprabhu S (2012). One-pot synthesis of conducting graphene-polymer composites and their strain sensing application. Nanoscale.

[22] Titelman GI, Gelman V, Bron S, Khalfin RL, Cohen Y, Bianco-Peled H (2005). Characteristics and microstructure of aqueous colloidal dispersions of graphite oxide. Carbon.

[23] Lane TJ, Nakagawa I, Walter JL, Kandathil AJ (1962). Infrared investigation of certain imidazole derivatives and their metal chelates. Inorg. Chem..

[24] Han MG, Foulger SH. 1-Dimensional structures of poly(3,4-ethylenedioxythiophene)(PEDOT): a chemical route to tubes, rods, thimbles, and belts. Chem. Commun*.* 2005**:**3092-3094 10.1039/b504727g 15959595

[25] McAllister MJ, Li JL, Adamson DH, Schniepp HC (2007). Single sheet functionalized graphene by oxidation and thermal expansion of graphite. Chem. Mater..

[26] Li K, Guo D, Lin F, Wei Y, Liu W, Kong Y (2015). Electrosorption of copper ions by poly(m-phenylenediamine)/reduced graphene oxide synthesized via a one-step in situ redox strategy. Electrochim. Acta.

[27] Zhao Q, Jamal R, Zhang L, Wang M, Abdiryim T (2014). The structure and properties of PEDOT synthesized by template-free solution method. Nanoscale Res Lett.

[28] Liu S, Tian J, Wang L, Luo Y, Lu W, Sun X (2011). Self-assembled graphene platelet–glucose oxidase nanostructures for glucose biosensing. Biosens. Bioelectron..

[29] Österholm A, Lindfors T, Kauppila J, Damlin P, Kvarnström C (2012). Electrochemical incorporation of graphene oxide into conducting polymer films. Electrochim. Acta.

[30] Lindfors T, Boeva ZA, Latonen R-M (2014). Electrochemical synthesis of poly(3,4-ethylenedioxythiophene) in aqueous dispersion of high porosity reduced graphene oxide. RSC Adv..

[31] Nie T, Zhang K, Xu J, Lu L, Bai L (2014). A facile one-pot strategy for the electrochemical synthesis of poly(3,4-ethylenedioxythiophene)/zirconia nanocomposite as an effective sensing platform for vitamins B2, B6 and C. J. Electroanal. Chem..

[32] Hohnholz D, MacDiarmid AG, Sarno DM, Jones JWE (2001). Uniform thin films of poly-3,4-ethylenedioxythiophene (PEDOT) prepared by in-situ deposition. Chem. Commun..

[33] Ali A, Jamal R, Shao W, Rahman A, Osman Y, Abdiryim T (2013). Structure and properties of solid-state synthesized poly(3,4-propylenedioxythiophene)/nano-ZnO composite. Prog. Nat. Sci..

[34] Abdiryim T, Ubul A, Jamal R, Xu F, Rahman A (2012). Electrochemical properties of the poly(3,4-ethylenedioxythiophene)/single-walled carbon nanotubes composite synthesized by solid-state heating method. Synth. Met..

[35] Abdiryim T, Jamal R, Zhao C, Awut T, Nurulla I (2010). Structure and properties of solid-state synthesized poly(3′,4′-ethylenedioxy-2,2′:5′,2″-terthiophene). Synth. Met..

[36] Wang W, Lei W, Yao T, Xia X, Huang W, Hao Q, Wang X (2013). One-pot synthesis of graphene/SnO2/PEDOT ternary electrode material for supercapacitors. Electrochim. Acta.

[37] Reza Nabid M, Tabatabaei Rezaei SJ, Zahra Hosseini S (2012). A novel template-free route to synthesis of poly(3,4-ethylenedioxythiophene) with fiber and sphere-like morphologies. Mater. Lett..

[38] Bai X, Hu X, Zhou S, Li L, Rohwerder M (2015). Controllable synthesis of leaflet-like poly (3,4-ethylenedioxythiophene)/single-walled carbon nanotube composites with microwave absorbing property. Compos. Sci. Technol..

[39] Hu X, Xu L (2000). Structure and properties of 3-alkoxy substituted polythiophene synthesized at low temperature. Polymer.

[40] Mi H, Zhang X, An S, Ye X, Yang S (2007). Microwave-assisted synthesis and electrochemical capacitance of polyaniline/multi-wall carbon nanotubes composite. Electrochem. Commun..

[41] Wang H, Hao Q, Yang X, Lu L, Wang X (2010). A nanostructured graphene/polyaniline hybrid material for supercapacitors. Nanoscale.

